# Advanced Research in Cardiovascular and Hemodynamic Monitoring

**DOI:** 10.3390/biomedicines14010046

**Published:** 2025-12-25

**Authors:** Deema Gichi, Scott T. Benken

**Affiliations:** Retzky College of Pharmacy, University of Illinois Chicago, Chicago, IL 60612, USA; gichid@uic.edu

## 1. Introduction

Notable advancements in cardiovascular and hemodynamic monitoring have emerged in recent years [1,2,3,4,5], reshaping the relationship between capturing physiological data and applying it to guide therapy in critical care settings. This Special Issue, titled “Advanced Research in Cardiovascular and Hemodynamic Monitoring”, brings together six original articles that provide valuable insight into additional advancements and shed light on future directions for improving the evaluation and, subsequently, the management of critically ill patients.

## 2. Contributions

In a prospective study (SkInShock), Kopp et al. (Contribution 1) examined whether transepidermal water loss (TEWL) could serve as a non-invasive adjunct diagnostic indicator of both fluid balance and vascular tone. While clinicians often assess skin turgor and mucous membranes as an estimate of fluid status [1], standardized, objective, and non-invasive measurements of skin function remain uncommon. This feasibility study investigated the use of a handheld Tewameter^®^ device to measure transcutaneous water evaporation daily at different skin sites in eight mechanically ventilated Intensive Care Unit (ICU) patients with either septic or cardiogenic shock. The 24 h skin water loss was calculated from direct TEWL data and compared with the standard estimation formula (6 mL/kg/day plus 20% per °C deviation from 37 °C). TEWL values were consistent across body regions except the forehead. TEWL-based skin water loss was significantly less than formula estimates (*p* < 0.01). These formulas overestimated losses at low TEWL and underestimated them at high TEWL. The authors also investigated the relationship between TEWL and vascular tone by measuring the systemic vascular resistance index (SVRI) concurrently using PiCCO^®^ technology. They found that absolute TEWL values did not reliably reflect vascular tone on an individual, continuous basis. However, interindividual normalization of TEWL data revealed a statistically significant negative correlation with SVRI, suggesting that higher skin water loss is associated with lower vascular tone. Despite ongoing debate regarding the relevance and usefulness of the skin vascular bed in shock patients [2], the authors demonstrated that TEWL measurement is indeed a feasible, simple, and non-invasive approach that can augment the understanding of insensible fluid loss and vasodilation when used in conjunction with established hemodynamic measures.

Noor et al. (Contribution 2) published a comprehensive review article appraising the growing role of point-of-care ultrasound (POCUS) in acute-care settings. The four different types of shock all result from either inadequate perfusion or abnormal blood flow distribution [3]. Hemodynamic monitoring in shock has evolved from reliance on static measures towards more dynamic ones. Static measures, such as central venous pressure (CVP) and pulmonary artery occlusion pressure (PAOP), offer partial insight into fluid responsiveness, while dynamic parameters, like stroke volume variation (SVV) and pulse pressure variation (PPV), allow for a better understanding of physiological responses to interventions [4]. This shift is adopted in the Surviving Sepsis Guidelines’ recommendation to guide fluid resuscitation [5]. While many tools and techniques currently exist to support these dynamic measures, the authors highlight POCUS as an essential bedside tool, emphasizing its accessibility and ability to reproduce real-time results for managing circulatory failure. Noor et al. describe an organized POCUS approach that integrates assessment of both systolic and diastolic cardiac function, volume status, and volume responsiveness to develop a comprehensive understanding of a patient’s hemodynamic profile. POCUS provides the ability to directly capture changes in preload, contractility, and ventricular interaction, allowing clinicians to adjust shock management therapy with greater confidence in real-time, through repeated assessment. The review effectively addresses the importance of standardized training and consistent interpretation for broader implementation. The authors conclude that POCUS should not be utilized as a standalone diagnostic test, but rather as an extension of the physical exam that provides real-time feedback about a patient’s response to therapy, ultimately supporting streamlined hemodynamic management.

Barańska-Pawełczak et al. (Contribution 3) investigated whether invasive hemodynamic data obtained through right heart catheterization (RHC) could better predict the response to cardiac resynchronization therapy (CRT) for patients with heart failure (HF). Current guideline recommendations for CRT depend largely on clinical status and non-invasive measures. Some of the non-invasive measures include QRS duration and morphology, particularly left bundle branch block (LBBB), reduced left ventricular ejection fraction (LVEF), and echocardiographic assessment of cardiac structure and function. Pulmonary arterial systolic pressure (sPAP) estimates from echocardiography are frequently used to assess PH in CRT candidates. Prior studies have shown that lower sPAP at the time of implantation is associated with a reduced risk of hospitalization or mortality [6]. However, regardless of meeting these criteria, some patients with HF do not demonstrate a favorable response after implantation. This may be explained by the fact that non-invasive criteria may not fully capture hemodynamic congestion and pulmonary vascular load, which could influence the response to CRT. Building on current knowledge, the authors proposed that augmenting existing CRT selection criteria with baseline invasive hemodynamic measurements may provide additional insight into post-implantation response. RHC enables the direct evaluation of cardiac output (CO), filling pressures, and pulmonary pressures, thereby highlighting the circulatory status within a broader physiological context. In addition to echocardiographic sPAP, the authors explored RHC-derived parameters, including right atrial pressure (RAP), pulmonary capillary wedge pressure (PCWP), and cardiac index (CI). The article showed that lower baseline left-sided filling pressures and a more favorable overall hemodynamic profile are characteristics of patients who respond positively to CRT. Furthermore, the study identified that lower transpulmonary gradient (TPG) and lower pulmonary vascular resistance (PVR) are meaningful indicators associated with CRT response. These findings are supported by existing data from Chatterjee et al. [7], which showed that patients with HF with TPG greater than 12 mmHg measured within six months before CRT implantation experienced significantly less improvement in their New York Heart Association (NYHA) functional class over a two-year period compared with those with lower TPG values. These findings suggest that a baseline invasive RHC assessment may help identify patients most likely to respond positively to CRT, supporting a more individualized approach to device utilization in addition to the established guideline recommendations.

The retrospective cohort study by Dalton et al. (Contribution 4) investigated whether end-tidal carbon dioxide (ETCO_2_) can be used as a real-time indicator of clinical deterioration. The study focused on mechanically ventilated ICU patients who experience in-hospital cardiac arrest (IHCA). Existing evidence established ETCO_2_ as a predictive marker in cardiac arrest, with lower ETCO_2_ levels associated with impaired CO. Furthermore, studies by Falk et al. [8] and Sanders et al. [9] demonstrated that ETCO_2_ values greater than 10 mmHg during resuscitation are associated with a higher likelihood of return of spontaneous circulation (ROSC). In line with this evidence, Dalton et al. took a step further and evaluated serial ETCO_2_ measurements for up to 48 h before IHCA and compared that to a 48 h time window after intubation for a control group of ICU patients that did not have cardiac arrest. Patients who had IHCA had significantly lower ETCO_2_ values leading up to the time of their cardiac arrest compared with a matched control group. The ETCO_2_ values were a mean of 20.0 mmHg in the IHCA cohort vs. 34.7 mmHg in the non-IHCA cohort (*p* < 0.001). Furthermore, the study showed that the difference was more pronounced as early as five hours immediately preceding the IHCA. The study also noted that ventilatory parameters and hemodynamic variables did not completely account for this decline, supporting prior evidence that ETCO_2_ mirrors changes in perfusion rather than ventilation alone. These findings support the use of ETCO_2_ as a non-invasive, continuous marker of CO and perfusion status. Overall, the authors emphasized the clinical utility of monitoring ETCO_2_ trends to aid clinicians in identifying high-risk patients before deterioration.

In another prospective study, Markus et al. (Contribution 5) assessed the hemodynamic and renal effects of mitral valve transcatheter edge-to-edge repair (MV-TEER) in patients with severe secondary mitral regurgitation (SMR) and advanced renal insufficiency. The study utilized non-invasive bioimpedance monitoring with NICaS^®^ before and after MV-TEER. NICaS^®^ is an FDA-approved bedside device that delivers accurate hemodynamic parameters, such as CO, stroke volume (SV), and systemic vascular resistance (SVR). Existing clinical trials by Paredes et al. [10] and Taniguchi et al. [11] have shown NICaS^®^ to be an effective tool. The use of NICaS^®^ allowed for continuous and radiation-free assessment of physiological changes in a population traditionally considered high-risk for invasive monitoring due to advanced renal dysfunction. Prior landmark trials, such as COAPT [12] and MITRA-FR [13], have established that reducing SMR can improve forward SV and further relieve congestion. Consistent with the existing evidence, Markus et al. demonstrated immediate and sustained improvements in cardiac performance following MV-TEER. These improvements include reductions in left atrial pressures and PH, as well as subsequent increases in CO and CI. Beyond cardiac improvement, patients also demonstrated measurable improvements in their renal function, as reflected by increases in estimated glomerular filtration rate (eGFR). This recovery suggests that some components of the cardiorenal syndrome may be reversible through unloading the left ventricle and improving forward flow. Additionally, improvements in NYHA functional class and quality-of-life measures were observed, emphasizing the clinical relevance of the hemodynamic changes captured by NICaS^®^. Overall, this study highlights the value of non-invasive hemodynamic monitoring and further supports its integration into the evaluation of treatment response and the guidance of management in complex MV-TEER patients.

The final article in this Special Issue is a comprehensive review by Galvez-Sánchez et al. (Contribution 6) that evaluates recent advancements in remote hemodynamic monitoring for patients with HF. This review highlights the utility of continuous physiological monitoring in enhancing the early recognition of decompensation signs and reducing hospitalization rates. The authors underscore that congestion and hemodynamic deterioration often precede clinical symptoms, sometimes by days to weeks. This creates a valuable opportunity for earlier intervention and a shift from reactive to proactive HF care. The authors compared established invasive monitoring systems, such as the CardioMEMS PAP sensor, with emerging non-invasive modalities. While CardioMEMS has been widely studied and has demonstrated up to a 28% reduction in HF hospitalization in the CHAMPION trial [14], the authors acknowledge the limitations of these invasive systems, including procedural risks, cost, and restricted patient eligibility. In parallel, the review highlights some of the growing non-invasive remote monitoring technologies, including wearable sensors, implantable loop technologies, and artificial intelligence (AI)-driven prediction models. Although these tools do not directly measure intracardiac pressures, they offer a low-risk, accessible, and longitudinal assessment of physiological trends in relation to volume status and cardiac performance. The non-invasive nature of these tools enables broader adoption across the HF population, supporting earlier intervention and outpatient management. Overall, the authors acknowledge the clinical utility of invasive monitoring, especially in high-risk patients, due to the precision of the data it provides. Simultaneously, they address the applicability of complementing the invasive approach with dynamic non-invasive monitoring tools to further optimize HF care. It is important to refine the predictive accuracy of AI models and ensure health equity and unbiased access to these rapidly advancing technologies.

## 3. Conclusions

In conclusion, this Special Issue summarizes the rapidly evolving landscape of cardiovascular and hemodynamic monitoring, highlighting a clear shift toward more dynamic, integrative, and personalized approaches in the management of critically ill patients. The included studies span the full spectrum from bedside non-invasive tools such as TEWL and POCUS, to invasive modalities like RHC, and advanced post-intervention assessments. Understanding and ultimately predicting the patient’s hemodynamic outcome requires integrating multimodal data, recognizing the limitations of each tool, and properly interpreting physiological signals.

## Abbreviations

AI	Artificial intelligence
CI	Cardiac index
CO	Cardiac output
CRT	Cardiac resynchronization therapy
CVP	Central venous pressure
eGFR	Estimated glomerular filtration rate
ETCO_2_	End-tidal carbon dioxide
FDA	U.S. Food and Drug Administration
HF	Heart failure
ICU	Intensive Care Unit
IHCA	In-hospital cardiac arrest
LBBB	Left bundle branch block
LVEF	Left ventricular ejection fraction
MV-TEER	Mitral valve transcatheter edge-to-edge repair
NYHA	New York Heart Association
PAOP	Pulmonary artery occlusion pressure
PAP	Pulmonary artery pressure
PCWP	Pulmonary capillary wedge pressure
PH	Pulmonary hypertension
PiCCO	Pulse contour cardiac output monitoring
POCUS	Point-of-care ultrasound
PPV	Pulse pressure variation
PVR	Pulmonary vascular resistance
RAP	Right atrial pressure
RHC	Right heart catheterization
ROSC	Return of spontaneous circulation
sPAP	Systolic pulmonary arterial pressure
SMR	Secondary mitral regurgitation
SV	Stroke volume
SVR	Systemic vascular resistance
SVRI	Systemic vascular resistance index
SVV	Stroke volume variation
TEWL	Transepidermal water loss
TPG	Transpulmonary gradient
